# Modern perspectives on psychoses: dissociation, automatism, and temporality across exogenous and endogenous dimensions

**DOI:** 10.3389/fpsyt.2025.1543673

**Published:** 2025-03-20

**Authors:** Valerio Ricci, Maria Celeste Ciavarella, Carlotta Marrangone, Guilherme Messas, Giuseppe Maina, Giovanni Martinotti

**Affiliations:** ^1^ San Luigi Gonzaga Hospital, University of Turin, Orbassano, Italy; ^2^ Department of Neurosciences, Imaging and Clinical Sciences, Università degli Studi G. D’Annunzio Chieti-Pescara, Chieti, Italy; ^3^ Department of Mental Health, Santa Casa de São Paulo School of Medical Sciences, Sao Paulo, Brazil; ^4^ Collaborating Centre for Values-based Practice in Health and Social Care, St Catherine’s College, Oxford, United Kingdom; ^5^ Department of Neurosciences “Rita Levi Montalcini”, University of Turin, Turin, Italy

**Keywords:** schizophrenia, novel psychoactive substances, cannabis, psychosis, mental automatism, dissociation, spaltung, exogenous psychosis

## Abstract

Substance use and the emergence of Novel Psychoactive Substances (NPS) present a significant public health challenge and diagnostic dilemma, particularly in the context of psychosis. The increasing availability of psychoactive substances among youth has led to a rise in Substance Use Disorders (SUDs), with profound implications for mental health. This paper explores the psychopathological distinctions between substance-induced psychoses (SIPs) and endogenous psychoses, such as schizophrenia, from a phenomenological perspective. We emphasize three key aspects: dissociation, mental automatism, and temporality, to elucidate the underlying mechanisms of these conditions. Dissociation, as a psychopathological organizer, is central to exogenous psychoses, particularly those triggered by NPS. This phenomenon leads to a fragmentation of consciousness, detachment from reality, and disintegration of identity, distinct from the spaltung observed in endogenous psychoses. The concept of mental automatism, as theorized by De Clerambault, is also explored, highlighting its role in the early stages of exogenous psychoses, where cognitive disruptions precede delusions and hallucinations. Furthermore, the temporal experience in SIPs is characterized by a disconnection from past and future, trapping individuals in an “eternal present.” This contrasts with the fragmented temporality observed in schizophrenia, where patients struggle to maintain a coherent narrative of their lives. The phenomenological approach provides critical insights into the clinical differentiation between SIPs and Primary Psychotic Disorders (PPDs), emphasizing the need for targeted interventions that address the specific temporal and cognitive disruptions in substance-induced conditions. In conclusion, this paper underscores the importance of integrating phenomenological psychopathology into clinical practice, particularly in the face of the evolving landscape of substance use and psychosis. Understanding the distinct psychopathological mechanisms underlying SIPs can inform more accurate diagnoses and effective treatments, ultimately improving patient outcomes in the context of this growing public health issue.

## 1 Substance use, psychosis, and the rise of novel psychoactive substances: a public health challenge and diagnostic dilemma

Psychoactive substances, both lawful and unlawful, are readily accessible and inexpensive for today’s youth. These substances offer quick and straightforward alterations in consciousness and emotional states in response to a dynamic environment. Substance Use Disorders (SUDs) are a significant public health issue, ranking among the top causes of years lived with disability worldwide in 2010 (1). Currently, substance use is so prevalent that it can hardly be considered deviant behavior, despite its severe implications for mortality rates and psychiatric burdens. The ethnopsychiatrist Devereux (2) posited that each society establishes its own norms and rules, shaping individuals’ expressions of illness/distress and success/fulfilment. In today’s society, substance use is intertwined with both these aspects. Stimulants are often viewed as enhancing successful lifestyles, promoting a sense of “empowerment” and reinforcing the narcissistic invulnerability seen in certain personality types. Conversely, substance use can create a perception of wholeness by simplifying life experiences and offering temporary relief from the overwhelming complexities of daily existence. For vulnerable individuals struggling with the demands of modern life, this may alleviate psychological distress and reduce feelings of inadequacy (3). In this context, substance use can be interpreted as a form of self-medication or a strategy for managing emotional discomfort (4). From this perspective, Substance Use Disorders (SUDs) and associated psychopathologies may be understood as culturally influenced phenomena, shaped by the fluid and ever-changing nature of contemporary social and cultural dynamics.

Beyond traditional psychoactive substances, which have become more potent, emerging concerns are directed toward Novel Psychoactive Substances (NPS), largely due to their rapid evolution and the virtually limitless online market (5–10). The European Union (EU) legally defines NPS as new narcotic or psychotropic substances, whether in pure form or preparations, not covered by the Single Convention on Narcotic Drugs of 1961 or the Convention on Psychotropic Substances of 1971 (11). Despite this, they present a public health risk potentially similar to substances listed in these conventions. By 2017, the European Union had identified over 670 NPS, with 632 identified since 2004 (12). These substances often go undetected by health professionals due to the lack of reliable, evidence-based information. The internet, including online forums, chat rooms, and blogs, has become a primary source of drug-related information, with approximately 61% of young Europeans aged 15-24 citing it as a key source of such information (13, 14). Within these platforms, users exchange experiences with various substances, recommend sources, and discuss administration methods. Vulnerable young individuals are particularly susceptible to aggressive marketing strategies employed by NPS vendors, such as attractive names, vibrant packaging, and free samples, increasing the likelihood of early substance exposure. Moreover, the largely unregulated nature of NPS contributes to their popularity, perpetuating the misconception of low associated risks (15, 16). Comprehensive surveys on the prevalence of NPS diffusion remain scarce, and evidence suggests that NPS consumption often occurs unintentionally (17, 18), apart from its use in prisons that appears to be quite popular and definitely intentional (19). Increasing evidence underscores the significant psychiatric and physical risks associated with NPS consumption. Initially, phenethylamines and tryptamines were the most common types of NPS, but in recent years, cathinones, synthetic cannabinoids (SC), phencyclidine, and benzofurans have gained popularity (20–22). Several NPS have been linked directly or indirectly to severe adverse effects and fatalities. For instance, 2-DPMP and D2PM, synthetic stimulants in the piperidine class, have shown neuropsychiatric and cardiovascular toxicity, contributing to three deaths in August 2010; misuse of gamma-hydroxybutyrate (GHB) and gamma-butyrolactone (GBL) has been associated with over 150 fatalities in the UK between 1995 and 2013 (23, 24). More recently, the emergence of novel synthetic opioids presents a significant concern, contributing to a rising number of overdose deaths in both the United States and Canada (25–27). The prevalence of Novel Psychoactive Substances (NPS) is notable among adolescents and young adults, particularly among psychiatric patients who report experiencing psychotic symptoms at the onset of their illness (28). Furthermore, substantial evidence suggests that NPS are a significant risk factor for violence and aggression among individuals diagnosed with major mental disorders (29–32).

Substance use frequently coexists with schizophrenia and other psychiatric conditions. The prevalence of Substance Use Disorder (SUD) among individuals with psychiatric illnesses is notably elevated, reaching up to 50%, a stark contrast to rates in the general population (33, 34). This high level of comorbidity poses significant challenges for clinicians and researchers in accurately distinguishing substance-induced psychopathology from primary psychiatric disorders within the context of concurrent SUDs (35, 36). According to the DSM-5, between 7% and 25% of individuals experiencing their first episode of psychosis exhibit symptoms of Substance-induced Psychosis (SIP). In a study conducted in Denmark, Norway, and Sweden, the annual incidence of SIP was estimated to be approximately 6.5 cases per 100,000 individuals, compared to 9.7 cases among those with a primary psychotic disorder (PPD) and comorbid substance use, and 24.1 cases among those with PPD alone. Individuals with chronic and heavy substance use, including cannabis, amphetamines, psychedelics, and cocaine, are at particularly elevated risk (35). There is growing recognition that substance use is linked to the onset of psychosis, which can manifest during substance use and persist even after withdrawal or cessation. Individuals experiencing SIP often seek urgent assistance, either by contacting law enforcement or ambulance services or by presenting themselves at emergency rooms, as they or their family and friends perceive an immediate need for intervention. Heavy and prolonged substance use can abruptly alter an individual’s perception and cognition, resulting in frightening experiences that may not be recognized as stemming from acute intoxication and necessitate urgent attention (37, 38). Some individuals may endure substance-induced psychotic symptoms for extended periods before seeking help. There is a dearth of research dedicated to differentiating Substance-Induced Psychosis (SIP) from Primary Psychotic Disorders (PPD), particularly in terms of elucidating potential divergent trajectories and outcomes (39–41). Limited understanding exists regarding factors specifically associated with SIP, and there are doubts regarding the efficacy of current classification systems in effectively distinguishing between primary and substance-induced disorders (42). Notably, the concept of substances inducing transient psychotic states was initially discussed in studies dating back to the 1960s. Several experimental studies have since corroborated this notion, demonstrating drug effects that mirror both the positive symptoms (e.g., hallucinations, delusions, paranoia, and disorganized thinking) and negative symptoms (e.g., affective flattening, anhedonia, attentional and cognitive impairment) characteristic of schizophrenia (43, 44). Of particular historical and phenomenological significance are the seminal self-experiments with mescaline conducted by Morselli and Huxley. Their detailed first-person accounts of psychedelic experiences represent foundational contributions to our understanding of how consciousness can expand into psychotic-dissociative dimensions. These pioneering investigators systematically documented their subjective experiences under mescaline, providing invaluable insights into the phenomenology of altered states and the boundaries of normal consciousness (45, 46). Current major neurobiological theories of schizophrenia trace their roots back to the effects of various substances: the serotonergic model stemmed from observations of lysergic acid diethylamide (LSD) (47), the dopamine hypothesis emerged from studies on amphetamines, and the glutamatergic model was influenced by research on phencyclidine (PCP) and ketamine (48–52). More recently, interest has grown in the role of endocannabinoids, spurred by the effects of cannabis (53–55). A multitude of substances have been implicated in the onset of psychosis, leading to the inclusion of Substance-Induced Psychosis (SIP) diagnoses in both the International Classification of Diseases 10 (ICD-10) (56) and the Diagnostic and Statistical Manual of Mental Disorders 5 (DSM-5) (57). The term SIP was first introduced in the fourth edition of the DSM in 1994, and its diagnostic criteria have remained largely unchanged in the DSM-5. SIP is characterized by the emergence of psychotic symptoms, such as hallucinations and delusions, during or shortly after substance intoxication or withdrawal, with symptoms resolving within a specified timeframe (1 month). The DSM-5 stipulates that psychotic symptoms in SIP should be more severe than those expected during intoxication or withdrawal and should necessitate healthcare intervention. Additionally, there should be a lack of insight into hallucinations and delusions. However, it is notable that this definition does not explicitly encompass negative symptoms, potentially overlooking a significant aspect of clinical presentation. In the ICD-10, the time criterion for Substance-Induced Psychosis (SIP) is slightly more stringent compared to the DSM-5, requiring partial resolution within one month and full resolution within six months. However, distinguishing between SIP and Primary Psychotic Disorders (PPD) remains a challenging diagnostic task in clinical practice. Misdiagnoses carry significant implications for clinical management, potentially leading to suboptimal follow-up and inappropriate treatment, with potential ramifications for prognosis.

In the DSM, a diagnosis of SIP hinges on the assumption that symptoms will dissipate following sustained abstinence. However, literature indicates that diagnostic changes over time are common, with transition rates from SIP to PPD ranging from 25% to 50% (58, 59). Recent research suggests that SIP is associated with a substantial risk of transitioning to schizophrenia, particularly following cannabis-induced psychosis (60, 61). These high transition rates are attributed partly to the progression of the psychotic disorder (SIP evolving into PPD) and partly to the narrowed definition of SIP, which may predispose to misdiagnosis (SIP being diagnosed as Schizophreniform Disorder or Psychosis NOS when schizophrenia criteria are not met) (62).

The aforementioned arguments raise some critical questions: what are the main psychopathological mechanisms underlying substance-induced psychoses that make them distinct from endogenous psychoses? The widespread use of new substances of abuse, the increased potency of traditional substances, and the corresponding increase in atypical forms of psychosis have brought this dramatic question to light. Furthermore, resolving this dilemma can lead to extensive discussions on the immediate recognition of these psychoses as well as appropriate treatment.

Given these premises, the aim of our work is to outlin the clinical characteristics of substance-induced psychoses in comparison to endogenous psychoses. Specifically, we will focus on three fundamental aspects: dissociation, mental automatism, and temporality. The specific points we will address are:

1. Describing the psychopathological organizers underlying the development of exogenous and endogenous psychoses, which we have identified as *dissociation* for the first group (63) and *spaltung* for the second group (64).
2. Defining mental automatism as a clinical phenomenon from which the psychotic symptoms typical of exogenous psychoses arise, serving as a precursor to Schneiderian first-rank symptoms (65).
3. Defining how the temporality of patients with exogenous psychoses develops in its clinical-existential essence.

For clarity and to avoid linguistic confusion, we will refer to the original terms, defining “dissociation” as the psychopathological condition related to exogenous psychoses and “spaltung” as that related to endogenous psychoses.

## 2 Dissociation: historical overview and the development of Janet’s theory in the context of substance-induced psychoses

Dissociation is a well-recognized psychological phenomenon where an individual feels detached from their thoughts, emotions, or environment. This often results in a disconnection from reality or disruptions in consciousness. The term “dissociation” covers several concepts. First, it acts as a defense mechanism, separating parts of an individual’s mental content from their conscious awareness while keeping these parts accessible. Unlike repression, which isolates only cognitive components, dissociation involves both cognitive and emotional elements, preventing access to both. Secondly, “dissociation” refers to various symptoms and phenomena with dissociative characteristics. These include derealization, depersonalization, dissociative amnesia, psychological numbing, absence phenomena, and the presence of distinct autonomous personalities within the same individual. Clearly, “dissociation” covers a range of symptoms characterized by altered states of consciousness, where individuals temporarily lose the continuity of their identity (66, 67). More specifically, we can distinguish between two fundamental categories of dissociative phenomena: detachment and compartmentalization. Detachment phenomena are characterized by an altered state of consciousness with a sense of separation from immediate experience, manifesting as depersonalization (separation from one’s body or mental processes), derealization (experiencing the external world as unreal), or ‘out-of-body’ experiences. In contrast, compartmentalization involves deficits in the ability to deliberately control normally controllable processes, manifesting as dissociative amnesia, conversion symptoms, or identity fragmentation. This distinction becomes particularly relevant when examining substance-induced versus endogenous conditions, as they tend to manifest different patterns of dissociative phenomena (66, 67).

Additionally, “dissociation” describes specific personality disorders marked by frequent dissociative symptoms and a lack of integration among different facets of an individual’s personality, experienced as distinct autonomous entities. This is particularly evident in dissociative identity disorder and more recent theoretical frameworks (68, 69). Lastly, “dissociation” involves phenomena that result in the fragmentation of specific functions associated with intrinsic biopsychological subsystems, such as attachment reactions and escape responses. It also includes mechanisms that drive individuals to avoid stimuli that might reactivate these subsystems, leading to behaviors resembling phobias and mental actions, ultimately reactivating dissociated contents with a traumatic origin linked to these subsystems (70, 71). Considering dissociation as the psychopathological organizer of exogenous psychoses, particularly those induced by the use of substances, requires a historical framework referencing Janet’s theories on dissociation. From this foundation, we can address exogenous psychoses, specifically substance-induced psychoses, which have distinct clinical and psychopathological characteristics compared to endogenous psychoses. To clarify this, we must distinguish between Janetian dissociation and Bleulerian “spaltung,” utilizing the significant contributions of De Clerambault in the psychogenesis of substance-induced psychoses. It is noteworthy that Bleuler’s use of spaltung postdates his introduction of the term “schizophrenia” in 1908 and is, in fact, linked to a specific conceptualization of psychotic splitting. Prior to coining “schizophrenia,” Bleuler employed the term “dissociation” to describe similar phenomena, acknowledging that these concepts were essentially synonymous. In this respect, spaltung and dissociation can be seen as overlapping phenomena, both describing a fragmentation of the psyche, though the term spaltung is more specific to the psychic disintegration associated with schizophrenia.

This analysis will highlight how dissociated consciousness serves as the psychopathological organizer of exogenous psychoses, disrupting the organization of temporality. We will focus on the subjective perception and experience of time continuity within the context of exogenous psychosis.

In contrast to psychoanalysis, which traditionally focused on concepts such as repression and internal conflict as the primary causes of hysteria, Pierre Janet offered a different perspective. He emphasized the importance of actual trauma in the development of dissociation and subsequent psychological disorders, diverging from Freud’s focus on “phantasmatic” trauma resulting from repression and childhood seduction (65, 72, 73). Modern understanding now acknowledges the significant impact of genuine traumatic experiences during development, including childhood relational trauma, abuse, maltreatment, and severe neglect. This recognition has reignited interest in studying dissociation across various pathological conditions, such as substance use disorders, borderline personality disorder, dissociative identity disorder, post-traumatic stress disorder (PTSD), and somatoform disorders. In Janet’s work “Automatisme psychologique” (65), he posits that dissociation and hysteria result from “psychological disaggregation” rather than the synthesis process essential to psychological well-being. Janet argued that certain symptoms exhibited by individuals with hysteria can be attributed to fragmented aspects of their personality that have become detached from consciousness. These fragments exist independently and originate from past traumatic events. Janet’s viewpoint assigns considerable importance to environmental trauma during an individual’s emotional and cognitive development. Unlike Freud’s early focus on sexual experiences, Janet believed this trauma has a fragmenting and disorganizing effect on an individual’s psychic activities. It hinders the formation of a conscious memory of the event, leading to “subconscious fixations” and “frozen emotions” that are rigidly excluded from personal consciousness, often resulting from sudden or terrifying shocks. If these traumatic memories are repeated during development or are exceptionally intense, they may solidify outside the individual’s conscious awareness, forming distinct secondary dissociated personalities, referred to as “successive existences.” These “fragments” can resurface abruptly and intensely. Another characteristic of hysterical disorders is the automatic and intrusive nature of the traumatic memories that contributed to their development. Janet proposes that dissociation, or “disintegration,” involves the disconnection of normally interconnected and integrated functional levels, a result of the intense emotions provoked by traumatic experiences.

Janet’s model draws on the psychological theories of Hughlings Jackson (74), who suggested that the mind results from the hierarchical integration of different functions, reflecting the evolutionary history of the species. It can be hypothesized that a childhood marked by recurring traumatic events and the repeated use of dissociative detachment processes impedes the progression of higher integrative functions. This hindrance, along with other pathogenic mechanisms associated with neurotoxic processes triggered by cumulative traumatic stress responses in critical structures like the hippocampus, cingulate gyrus, frontal lobes, and insula, may weaken the higher functions of consciousness (75). This impairment might prevent the ability to integrate experiences, leading to the fragmentation of autobiographical memories and the sense of self. Following Jackson’s hierarchical model, substance-induced (exogenous) psychoses typically manifest through detachment phenomena, particularly in early stages, while preserving basic functional hierarchies and disrupting only higher-order integration. This pattern aligns with the observed clinical presentation where individuals maintain basic functioning while experiencing significant alterations in consciousness and temporal experience. In contrast, endogenous conditions more commonly feature compartmentalization phenomena, reflecting a deeper disruption of functional hierarchies and corresponding to the more profound fragmentation of self-experience seen in schizophrenia. This distinction helps explain the different recovery trajectories: exogenous psychoses often show better recovery potential once the triggering substance is removed, as the basic hierarchical integration remains intact, while endogenous conditions, involving more fundamental compartmentalization, typically require more comprehensive therapeutic approaches. The Jacksonian theoretical framework elucidates Henri Ey’s organodynamic theory, providing interpretative keys for understanding psychotic mechanisms, particularly in exogenous psychoses. The organodynamic model postulates a hierarchical organization of consciousness, wherein toxic substances can induce a dissolution of higher integrative functions while preserving more elementary operations. This model clarifies why substance-induced experiences frequently maintain basic reality testing even during acute phases of psychotic episodes (76).

Janetian concepts of dissociation have sparked increasing interest in exploring the complex relationship between substance abuse and dissociation, leading to the development of evolving research lines. The etiology of pathological addiction is now acknowledged as multifactorial, involving interactions between neurobiological, psychological, and socio-environmental dimensions (77). Various explanatory models have emerged to understand the connection between trauma, dissociation, and addiction. According to the self-medication hypothesis, addiction can be viewed as a maladaptive attempt to address fundamental disturbances in self-regulation, self-consistency, relationships, and self-care (78, 79). This model posits that deficits in attachment relationships, particularly insecure or disorganized/unspecified attachments, combined with childhood traumatic experiences, may result in an inability to recognize and regulate emotional states. Individuals may turn to dissociative defense mechanisms, such as substance use or addictive behaviors, to cope with internal distress. This creates a psychobiological vulnerability to addiction, establishing it as a preferred strategy for managing intolerable internal states by seeking pleasure or alleviating pain, with a tendency toward reliance on substances or addictive behavior (80, 81).

In this context, Olievenstein, a disciple of Lacan, identifies a specific psychological mechanism to describe the genesis of addiction: the theory of the “broken mirror phase.” In this theory, a primary traumatic fracture triggers the patient’s continuous search for the substance, aiming to re-experience, through the substance itself, the initial successful encounter with the Self’s image. The substance temporarily takes the place of the fracture and erases it, explaining the inherent inability of the drug-dependent individual to remain in a state of quietness and their potential to engage in a cohesive encounter with the passage of experiential time (82).

A potential biological basis for this relationship may involve alterations in the response to reward, affiliative behaviors, and stress responses, sensitive to different types of traumatic experiences (83). Patients affected by both post-traumatic conditions and addiction often exhibit common distress patterns arising from the correlation between insecure attachment and the presence of childhood trauma (84, 85). Concerning dissociative psychopathology in opioid use disorder, Somer et al. (86) introduced the concept of ‘chemical dissociation,’ suggesting that some substance-abusing patients may not exhibit high levels of dissociation despite their trauma history, as they may achieve dissociative-like states through chemical consumption to manage overwhelming emotional states. The substances themselves may also decrease tolerance to painful states, reinforcing substance use and the persistence of addiction. Recent studies have explored the role of PTSD in individuals with substance abuse (87, 88), but fewer investigations have specifically addressed the comorbidity of dissociation, a common consequence of early trauma (89, 90). Current findings indicate that this type of psychopathology is associated with a more severe course of the disorder, additional complications (e.g., suicidality and self-mutilation) (91, 92), and a negative impact on treatment outcomes (93). Higher levels of dissociative symptoms and a greater prevalence of dissociative disorders have been observed in individuals with drug addiction and both alcohol and drug dependence, compared to those with pure alcoholism (94). Using structured clinical interviews with tools such as the Structured Clinical Interview for Dissociative Disorders, Tamar-Gurol (95) demonstrated that 26% of the substance-dependent patients in their study had a dissociative disorder. Among these patients, the majority (59.3%) reported that the dissociative disorder preceded the onset of substance abuse. This finding complicates the ability to differentiate between the dissociative effects of the substance itself and its use as a coping mechanism for emotional distress. Even before substance use, the capacity to recognize the need for employing dissociative defenses appears compromised.

### 2.1 Dissociation as a psychopathological organizer in exogenous psychoses: contrasting Janet’s framework with Bleulerian spaltung

Examining Janet’s framework of dissociation, this study explores its role as a psychopathological organizer in substance-induced psychoses, referred to here as “exogenous psychoses,” inspired by Bonhoeffer’s significant insights (96). The exogenous theory of psychosis challenged the prevailing endogenous theory in European psychiatry from Kraepelin to Bleuler. Moreau de Tours sought to replicate psychotic symptoms similar to those seen in classic schizophrenia using various substances (97). The exogenous psychosis model, characterized by dissociation distinct from the schizophrenic spaltung process proposed by Bleuler, instead emerged as a disruption of consciousness induced by external factors. This concept reached its peak with Bonhoeffer, who practiced in Germany during the first half of the last century. Bonhoeffer is well-known for his descriptions of major psychopathological syndromes in conditions such as alcoholism and depression, particularly for his doctrine of the “exogenous type of reaction.” According to this doctrine, any harmful exogenous agent (infectious, toxic, traumatic, degenerative) acting on the brain generates a relatively uniform, nonspecific set of symptoms, with a mandatory disturbance of consciousness ranging from confusion to delirium, stupor, or coma. Bonhoeffer introduced the idea of exogenous reaction types as the brain’s response to injury. Since he could not establish a direct link between a specific trauma or toxin and the resulting mental disorder, he hypothesized the existence of intermediate products formed in the body that caused conditions such as delirium, twilight and confusional states, hallucinosis, and Korsakoff syndrome. This approach necessitates precise delineation of the scope of symptomatic psychoses. The concept of Spaltung theorized by Bleuler (64) refers to Karl Wernicke’s concept of Sejunktion (98), understood as the loosening of associations which, by generating false ideas, compromises the structure of the self and constitutes the key to all schizophrenic disorders. The destructuring process induced by Spaltung can have varying degrees of severity, reaching a maximum level of severity defined as Zerspaltung. If we draw an analogy, we can imagine a sphere in which the dissociation of exogenous psychoses is confined to the outer surface, not entirely altering the eidetic foundation of patients but disrupting internal connections and communications between various affective planes (as hypothesized by Janet). Spaltung, on the other hand, is a fissure or split that fractures the sphere toward the center. Spaltung is a unique, stable concept that either exists or does not. The dissociation in exogenous psychoses has a more dynamic quality; it oscillates between phases of twilight states with psychotic onset and phases of restored perception of reality.

Bleuler was aware of the confusion that the term spaltung (64) could sometimes cause with the term *Dissociation* (and we cannot deny that Bleuler inherited this term from the French tradition); for this reason, Bleuler himself referred to zerspaltung, giving it a greater degree of severity in the structure of endogenous schizophrenia, particularly in a prognostic sense. Spaltung in schizophrenics indicates the rupture of syntactic and semantic connections between various psychic representations and concepts: from prosaic metonymies to more poetic metaphors. Logic and realism disappear. Like many of his contemporaries trained in the idealistic school, Bleuler assumed the existence of a normally unitary psychic entity, a subjective essence, the psyche, which shatters in illness.

If in the schizophrenic psyche the unifying principle between the individual complexes, which do not communicate with each other, is missing, in those with exogenous psychoses this principle is preserved, at least in the early stages. For this reason, the element of “goal-directed representation,” which stably situates the subject within their social context in agreement with the goal-directed representations of their peers and the collective in which the individual lives, and the agreement with the “generalized other,” is preserved in exogenous psychoses (99). This preservation avoids the isolation of the self from itself—spaltung—and from others—autism, which is typical of the phenomenology of endogenous schizophrenic psychosis.

In conclusion, further clarifying Bleuler’s distinctions between spaltung and autism helps elucidate their different roles in exogenous versus endogenous conditions. While Spaltung represents a structural fragmentation of psychic functions, autism manifests as a more global alteration in the patient’s way of being in the world. Spaltung primarily affects the logical connections of thought and language, preserving some basic reality-testing abilities, particularly in exogenous conditions. In contrast, autism represents a more fundamental withdrawal from shared reality, characterized by what Bleuler termed dereistic thinking—a mode of thought governed by desires and fears rather than logic, a structural model that Bleuler had identified in patients with schizophrenia simplex, where only the generative disturbance was evident (64). This differentiation, which should not be seen as overly rigid but rather as involving mutual correlations of meaning, provides crucial diagnostic indicators: exogenous psychoses typically maintain the capacity for goal-directed representation within their social context, whereas endogenous conditions manifest a more profound isolation of the self—from both itself (spaltung) and others (autism).

## 3 De Clerambault’s mental automatism in the psychopathological basis of psychosis

To explore psychopathological frameworks, we turn to the second essential component for understanding exogenous psychoses: the concept of mental automatism as theorized by De Clérambault, comparing and contrasting it with Kurt Schneider’s first-rank symptoms (100) Gaëtan Gatian de Clérambault (1872–1934), a prominent French psychiatrist, began his career in 1905 at the Special Infirmary for the Insane of the Paris Prefecture of Police, later becoming its head from 1920 to 1934. This facility, housing 18 cells, was designated for the temporary detention and psychiatric evaluation of individuals deemed potentially insane post-arrest (101). Each year, approximately 2,500 to 3,000 individuals were assessed here. De Clérambault was noted for his rapid yet thorough clinical assessments, concentrating on identifying key symptoms rather than solely diagnosing specific disorders. Over his career, he is believed to have issued between 13,000 and 15,000 certificates. In addition to his diagnostic work, he also engaged in teaching, often through public clinical presentations discussing selected cases (99).

De Clérambault’s study of mental automatism began with a brief paper in 1909 and evolved throughout his career, with significant recognition at the 1927 conference of Aliénistes et Neurologistes de France. He first explored mental automatism in depth in 1920, defining it by 1925 as “a clinical syndrome comprising automatic phenomena in motor, sensory, and ideo-verbal domains” (102). These phenomena refer to unexpected occurrences in one’s body or mind, perceived as foreign by the individual, leading to a passive experience of these intrusive elements, resulting in a sense of misappropriation and interference (103).

Initially surprising due to their spontaneous and mechanical nature, these phenomena remain emotionally neutral. The confusion and uncertainty stem from disruptions in the ideation process rather than specific mental content. De Clérambault stressed that mental automatism is not inherently hostile; the phenomena are emotionally neutral and lack thematic content initially (104).

He viewed mental automatism as a fundamental process underlying various mental illnesses, encompassing all known types of hallucinations; however, the concept extends beyond mere hallucinations (104). While including hallucinations, mental automatism indicates a general interference process affecting motor, sensory-affective, or ideo-verbal functions, often observed in early psychotic states (103, 104). Non-hallucinatory mental automatism typically precedes the onset of hallucinations, though both may coexist. Within this overarching interference process, de Clérambault identified two categories of automatic phenomena: positive and negative. Positive mental automatism involves the introduction of new elements into one’s functioning, while negative mental automatism pertains to the deterioration of habitual elements. The former involves intrusion phenomena, and the latter involves inhibition phenomena.

Throughout his work, de Clérambault distinguished between minor automatism (petit automatisme) and major automatism (grand automatisme). Minor automatism refers to subtle positive and negative ideo-verbal phenomena that have yet to significantly impact an individual’s functioning and often go unnoticed due to their lack of specific content and emotional neutrality. Major automatism involves sensory-affective and motor functions, where prominent automatic phenomena, including ideo-verbal ones, disrupt an individual’s functioning, leading to feelings of confusion and overwhelm. Minor automatism often precedes major automatism, though this is not always the case. Thought echoes may emerge during the transition between these experiences (104). De Clérambault’s approach diverged from conventional diagnostic systems that focused primarily on specific symptoms like hallucinations and delusions, instead offering a broader descriptive approach centered on mental automatism as a fundamental mechanism underlying various forms of psychosis. Traditional diagnostic methods aimed to provide an overall clinical picture of different psychotic conditions, while de Clérambault believed not all symptoms carried equal diagnostic weight. He sought to identify the elemental characteristic defining psychosis. According to his theory, patients experiencing hallucinations without automatic phenomena were not considered psychotic, whereas those experiencing automatic phenomena without hallucinations and delusions were classified as psychotic. In developing his theory of mental automatism, de Clérambault drew from the works of his predecessors in French psychiatry, notably Jules Baillarger’s ideas on thought echoes and psychic hallucinations (105) and Jules Séglas’s work on verbal psychomotor hallucinations (106), both addressing the experience of imposed thoughts or speech. De Clérambault’s clinical syndrome uniquely grouped these phenomena with a range of other intrusive experiences exerting similar effects on patients.

Regarding psychosis subtypes, de Clérambault associated mental automatism with various forms of paranoia, hypochondria, mania, melancholia, and hallucinatory psychoses. Despite these associations, he maintained a unified concept of psychosis, suggesting similar automatic phenomena could lead to different types of delusions. For instance, cenesthetic sensations could give rise to hypochondria or delusions with mystical or persecutory themes. Central to de Clérambault’s work was his belief in a direct correspondence between observed clinical manifestations of mental automatism and underlying neurological disturbances responsible for these phenomena. He adhered strictly to an organicist perspective, positing that mental automatism resulted from negative reactions to factors like infection, intoxication, or tumors. Given the mechanistic logic of automatism, he reasoned its origins must be mechanical, not psychological. De Clérambault dismissed psychological explanations as highly implausible and seemed indifferent to causation questions, using ‘mechanism’ perhaps more metaphorically (107). According to his theory, mental automatism is the primary mechanism of psychosis, with other symptoms, such as delusions, being secondary reactions. He described delusions as necessary responses of an intact reasoning intellect to subconscious phenomena. Delusions are expansions of automatic phenomena that overwhelm and disturb the individual: “the intensity, unexpected nature, constancy, and strangeness of the sensation lead the subject to seek an external explanation” (108). Delusional interpretations are cognitive responses that may eventually lead to the development of a “second,” delusional personality. Between the onset of automatic phenomena and the formation of a delusion, an “incubation period” may occur, during which the initial experience of intrusion gradually permeates the patient’s broader mental life. This period is characterized by confusion due to conflicting thoughts and experiences: “an unexpected image arises, provoking an irrefutable thought; then it becomes haunting, provoking several contradictory thoughts” (109). De Clérambault assigned a different significance to the productive symptomatology of psychoses, such as the concept of hallucinations traditionally defined, based on a long 19th-century tradition, as “perceptions without objects.” Within the context of this still problematic differentiation, De Clérambault’s work, through his theory of mental automatism, seeks to acknowledge the clinical value of so-called psychic phenomena. He emphasizes the “non-sensory” nature of automatism, stating that “thoughts become foreign in the ordinary form of thought, that is, in an undifferentiated form rather than a defined sensory form. The undifferentiated form consists of a mixture of abstractions and tendencies, either without sensory elements or with vague and fragmentary multisensory elements.” He further adds that it is “an autonomous process often found in isolation and does not inherently imply any delusion, although a delusion may appear many years after its onset.” De Clérambault describes eidetic automatism as a disruption of thought that may include intrusive thoughts, imposed thoughts, and anticipation of thoughts. These phenomena constitute an “Echo of thought,” where the thought process is duplicated in time and space without the patient initially feeling particularly affected or persecuted, and often in the absence of delusion.

These characteristics are particularly relevant in clinical practice, as some patients exhibit similar phenomena that appear to exist independently of their underlying clinical condition. These phenomena can emerge at the onset or during the course of treatment, sometimes recognized by the patients as obstacles to therapy, and other times noticed only by the clinicians during interviews. What is particularly intriguing is the structural nature of these phenomena. In mental automatism, there is an element that seems to function autonomously, representing a prelude that can culminate in delusional crystallization, which can be seen as a core feature of substance-related psychosis (4).

### 3.1 Mental automatism and dissociative nucleus in the exogenous paradigm

De Clérambault’s work highlights a dissociative nucleus that acts as a discerning observer of cascading automatic phenomena (107). This nucleus serves as the foundation for the emergence of secondary structures within the patient’s experience, such as delusional thoughts and sensory disturbances, including tactile, visual, and auditory hallucinations. His research elucidates the complex interplay between dissociation, automatic phenomena, and resulting psychopathological manifestations. This theoretical perspective introduces a new discourse around the concept of “exogenous,” a term historically overshadowed by the “endogenous” paradigm. Initially, “endogenous” implied hereditary influences and was often linked to degenerative processes, suggesting that certain disorders were due to inherent factors transmitted across generations, leading to progressive decline.

The theories of Morel and Magnan (110, 111) supported the notion of degeneration as central to endogenous disorders. However, Bonhoeffer provided a new perspective, arguing that distinguishing between exogenous and endogenous factors was not straightforward (96). He recognized the complexity of psychiatric disorders and the likelihood of mixed etiologies, blurring the lines between exogenous and endogenous clinical features. Bonhoeffer posited that pure forms of exogenous and endogenous etiology are rare and that a clear and exhaustive differentiation between the two is challenging. This perspective acknowledges the intricate nature of mental health disorders, influenced by environmental stressors, trauma, genetic predisposition, and neurobiological mechanisms. It advocates for a comprehensive understanding of the various factors influencing mental well-being, transcending rigid categorizations of exogenous versus endogenous factors. Bonhoeffer’s assertions introduce the concept of external pathogenic agents and the formation of dissociative psychosis on a heteroplastic foundation, challenging the longstanding discourse surrounding endogenous versus exogenous factors.

### 3.2 De Clérambault’s automatism vs. Schneider’s first-rank symptoms: different paradigms in endogenous and exogenous psychoses

The concept of mental automatism has deep roots in the broader psychopathological issue of Xenopathy, introduced by Giraud (112), which has significantly influenced subsequent theories, particularly Kurt Schneider’s concept of passivity in his first-rank symptoms (99). Xenopathy, a term combining “xenos” (foreigner) and “pathos” (disease), echoes xenophobia, commonly used to describe the fear of foreign populations and migrants. A synonymous term for xenopathy is “influence syndrome,” wherein the individual fails to recognize ownership of their words, actions, or movements, which are perceived as being influenced or imposed by an external force. Xenopathy refers to the sensation of being influenced by an external agent. Notably, Tausk’s “influencing machine” exemplifies this concept (113). De Clérambault highlighted the mechanistic nature of human linguistic organization through his notion of mental automatism, emphasizing the phenomenon of thought echo. In these instances, individuals feel that their most intimate thoughts are spied upon and mirrored by a continuous parallel auditory emission, effectively vocalizing their thoughts. De Clérambault conceptualized xenopathy as the foundation of chronic delusional syndrome. Those affected by this syndrome experience a range of elementary phenomena—motor, sensory, and cognitive—that, despite originating within their consciousness, are disconnected from their will. These phenomena appear inevitable, uncontrollable, repetitive, and unrelated to anything else, creating the sensation that their internal world is disrupted by autonomous elements. These automatic phenomena are characterized by their autonomy, primitiveness, and neutrality, which de Clérambault considered to have an organic basis. The term “automatisme mental” signifies a broad concept that is not uniformly understood but is recognized for its “organic” and “primary” nature. From a psychopathological perspective, mental automatisms, particularly when described as “pathological,” are seen as mechanisms that liberate psychic elements from the ego without the individual’s awareness and as conscious feelings of automatism that the person does not recognize as pathological. Drawing on Jacksonian theories, de Clérambault believed that intoxications could release activities typically controlled by higher inhibitory centers, resulting in autonomous, seemingly spontaneous automatic phenomena. De Clérambault asserted that these mental automatisms had a significant organic, biological component, with the patient’s critical ego passively observing the automatism, overlaid by delusion.

Kurt Schneider’s construct (100) was developed after the contribution of de Clérambault, and builds on Jaspers’ notion of mental automatism as a disturbance of ego consciousness. Schneider described it as a disruption in the sense of belonging to oneself, a mechanistic, parasitic syndrome deeply affecting the patient’s internal world. Schneider’s first-rank symptoms and de Clérambault’s concept of mental automatism both address disruptions in the individual’s sense of self and agency, but in distinct ways. Schneider’s first-rank symptoms, indicative of schizophrenia, include thought insertion, thought withdrawal, thought broadcasting, and auditory hallucinations commenting on one’s actions or conversing with each other. These symptoms reflect a profound intrusion into the individual’s mental life, suggesting a breakdown in the boundary between self and external reality. In contrast, de Clérambault’s mental automatism encompasses a broader range of involuntary phenomena, including motor, sensory, and cognitive elements. While both frameworks describe experiences disconnected from the individual’s will, mental automatism is more mechanistic and parasitic, emphasizing the autonomous nature of these phenomena as they disrupt the individual’s internal world. De Clérambault focused on the biological and organic underpinnings of these automatisms, viewing them as manifestations of a deeper physiological process. The primary similarity between the two concepts is their focus on experiences perceived as being imposed upon the individual, leading to a sense of alienation from their thoughts and actions. Both sets of symptoms underscore the disruption of normal self-experience and the emergence of involuntary, intrusive phenomena that challenge the individual’s sense of control and coherence. However, a key difference lies in the scope and specificity of the symptoms. Schneider’s first-rank symptoms are primarily centered on disturbances in thought and perception. In contrast, de Clérambault’s mental automatism is a broader construct that can encompass various involuntary phenomena across different sensory and cognitive domains, often seen in the context of chronic hallucinatory delusions and substance-induced psychoses.

The relationship between mental automatism and first-rank symptoms leads us to consider a more fundamental aspect in differentiating endogenous from exogenous psychoses: the role of basic self-disorders (SD) Recent meta-analytic evidence shows that SD selectively aggregate within schizophrenia spectrum disorders compared to other mental disorders and healthy controls (114). At their core, these disorders represent a fundamental disturbance of ipseity - the basic, pre-reflective sense of self-presence and first-person perspective that normally permeates all experience. This ipseity disturbance manifests as a profound alteration in the structure of consciousness, where the basic sense of being a unified, vital, and self-coinciding subject of experience becomes unstable or compromised. In schizophrenia spectrum disorders, this disturbance of ipseity typically involves three interconnected aspects: diminished self-presence (a weakened sense of existing as a vital and self-coinciding subject), hyperreflexivity (exaggerated self-consciousness where aspects of oneself that are normally implicit become explicit objects of awareness), and disturbed grip or hold on the world (loss of natural, immediate immersion in the shared world). These disturbances of basic self-experience appear to be specifically characteristic of the schizophrenia spectrum, representing a more fundamental alteration than the phenomenology of mental automatism seen in substance-induced states. While mental automatism describes the mechanism through which substances may trigger psychotic experiences through a temporary disruption of cognitive and perceptual processes, SD represent a more pervasive and enduring alteration in the basic structure of consciousness. This distinction raises an important research question: could the presence of pre-existing ipseity disturbance influence the trajectory of substance-induced psychoses? While no studies have directly compared SD (as measured by the Examination of Anomalous Self-Experience - EASE) (115), between schizophrenia and substance-induced psychosis patients, one could hypothesize that individuals who exhibit fundamental disturbances of ipseity and experience substance-induced psychosis may be at higher risk for developing chronic psychotic disorders (116, 117) This potential interaction between ipseity disturbance and substance-induced psychotic experiences suggests a more complex pathway from disruption of minimal self to anomalies in ego boundaries and ultimately to first-rank symptoms. The presence of basic self-disorders might represent a vulnerability factor that could influence how substance-induced alterations of consciousness evolve over time (118).

Further research is needed to explore whether assessment of ipseity disturbance could help predict differential outcomes in substance-induced psychoses and clarify the relationship between endogenous and exogenous pathways to psychosis.

In summary, while both Schneider’s first-rank symptoms and de Clérambault’s mental automatism describe significant disruptions in self-experience and agency, they represent different levels of psychopathological alterations. Schneider’s symptoms are more narrowly defined and tied to schizophrenia, reflecting a profound disruption of ipseity that characterizes the schizophrenia spectrum. In contrast, de Clérambault’s mental automatism provides a broader, more mechanistic perspective on involuntary phenomena within the mind, particularly relevant for understanding substance-induced psychotic experiences. This distinction becomes crucial when considering the role of basic self-disorders: while mental automatism may characterize the phenomenology of exogenous psychoses through temporary disruptions of cognitive and perceptual processes, the presence of fundamental ipseity disturbance might represent a core feature that distinguishes endogenous schizophrenia spectrum conditions from substance-induced states.

## 4 Temporality

The phenomenological exploration of the relationship between substance use and psychosis reveals distinct temporal divergences between endogenous and exogenous psychoses. Endogenous psychoses are primarily characterized by an internal temporal disruption, originating from within the individual’s psyche and not directly caused by external factors. In schizophrenia, there is a noticeable temporal fragmentation of self-experience (119). This disruption often leads to a disintegration of the individual’s temporal structure, including the disarticulation of past, present, and future experiences, resulting in a fragmented sense of time. This biographical instability causes events and experiences to lose their coherent temporal sequence, making it challenging for individuals to form a continuous narrative of their lives. Schizophrenic patients often struggle to articulate their self-narratives, losing the transcendence that makes human relationships unique; their intentional arc fragments, placing them in a desynchronized and fragmented temporal dimension (120–123). In pre-psychotic forms and at the onset of psychosis, where a profound anxiety of an impending world-ending catastrophe prevails, time appears to accelerate in an attempt to grasp the future and escape imminent disaster. This can be likened to an ante-festum perception (124) or Conrad’s concept of “trema” (125). As psychosis consolidates and becomes chronic, along with the loss of self-transcendence and the development of delusions, both implicit and explicit time become fragmented (126). In this context, the delusion serves a fundamental purpose: to reconstruct a temporal biographical narrative. However, the delusion confines the patient within a desynchronized and non-intersubjective time. The patient becomes passive to the influences of others, devoid of initiative, and isolated on an island outside of time (127). The origin of endogenous psychoses is primarily internal, linked to the individual’s inherent psychological and existential vulnerabilities, making the psychotic episodes more resistant to external interventions.

In contrast, exogenous psychoses are characterized by temporal fragmentation induced by external agents, altering the individual’s perception and experience of time. Temporal experiences in exogenous psychoses often become cyclical or repetitive, unlike the fragmented temporality in endogenous psychoses. This fragmentation traps the patient in the infinite moment of substance intoxication, forcing them to live in an eternal present, in a continuous state of intrafestum (124). The present moment, becomes overwhelmingly dominant, overshadowing past and future considerations. This condition is a condensation of reality, an intensification of the present moment that erases waiting time. This “continuous present” dominates the scene and disrupts the pre-reflective experience of time (4). As a result, the temporal horizon, which provides a background for imagining the future, contracts, and the future becomes both actualized and dissociated (127). The lived future, which typically encompasses aspirations and plans, loses its continuity with the present, leading to a narrowing of temporal distances and the collapse of all experiences into a single, infinite present. The term “Anthropological Hyperpresentification” describes the narrowing of temporality solely to the present, devoid of a connection to the past and future in conscious awareness (3, 128). Based on these premises, it can be speculated that there is a dissociation of consciousness or, more specifically, a dissociation of the future and an endless present. Aspirations and projects become detached from the present moment, which expands and becomes eternal, while the future loses its contextual framework, and aspirations lose continuity with the present (129–134).

In summary, while the temporal arc in endogenous psychoses is fragmented at multiple points, in exogenous psychoses, it is concentrated at a single point, namely the intoxication that triggers psychosis, leading to a pathological condensation of the experience, which works as a psychosis without a complete break from reality. However, this tends to reestablish itself after the substance’s effects wane, unless the continuous and massive use of the substance leads to a transition to persistent psychosis (see **Table 1**).

**Table 1:** This table compares the characteristics of *Spaltung* and *Dissociation* as central psychopathological mechanisms in endogenous and exogenous psychoses, respectively.

Characteristic	Endogenous Psychoses (Spaltung)	Exogenous Psychoses (Dissociation)
**Term Origin**	Derived from Bleuler, used to describe the psychic disintegration typical of schizophrenia	Derived from Janet, referring to the fragmentation of consciousness and disconnection linked to traumatic factors
**Definition**	Splitting of the psyche involving a stable and profound separation between internal psychic complexes	Temporary and dynamic disaggregation of consciousness and psychic functions in response to external factors
**Mechanism**	A stable fracture towards the center of the psyche, with loss of semantic and syntactic connections	Oscillation between dissociative states and partial reintegration of consciousness
**Clinical Manifestations**	Breakdown of logical associations, emergence of false ideas, and isolation from reality (autism)	Twilight states, confusion, and loss of contact with reality during intoxication
**Role of temporality**	Internal fragmentation of temporal continuity, with a loss of coherent biographical narrative.	Temporal condensation into an "eternal present" tied to the effects of substances.
**Psychopathological organizers**	A deep rupture compromising interaction between internal psychic representations and concepts	Dissociation as a response to trauma or substances temporarily disrupting integrative functions
**Prognosis**	Stability of the split, often associated with poor prognosis in chronic forms	Potential recovery with reduction or cessation of substance use, unless prolonged or massive exposure occurs
**Clinical examples**	Typical of schizophrenia, with cognitive and behavioral disorganization phenomena	Substance-induced psychoses, with reversible symptoms in the absence of prolonged exposure

It highlights their origins, definitions, mechanisms, clinical manifestations, temporal dynamics, and prognostic implications, providing a clear differentiation between the psychic splitting in schizophrenia and the dissociative processes observed in substance-induced psychoses.

## 5 Conclusions and clinical implications

The investigation of substance-induced psychoses and their differentiation from primary psychotic disorders (PPDs) has yielded substantial insights into the underlying psychopathological mechanisms, clinical manifestations, and treatment implications. Our findings confirm that dissociation is a critical psychopathological organizer in exogenous psychoses, particularly those induced by Novel Psychoactive Substances and high potency traditional substances. Dissociation, as defined by Janet, involves a detachment from cognitive processes, emotions, or the environment, leading to a fragmentation of the individual’s sense of reality and identity. This concept is distinct from Bleulerian spaltung which refers to the fundamental disintegration observed in endogenous psychoses like schizophrenia. Janet’s historical framework of dissociation underscores its role in the development of psychotic states following traumatic experiences. Our analysis supports the idea that dissociation underlies the abrupt changes in consciousness and identity seen in exogenous psychoses. The detachment and fragmentation experienced by individuals using psychoactive substances can be traced to traumatic environmental influences and the neurotoxic effects of these substances, which disrupt the higher integrative functions of consciousness. The resurgence of dissociation as a central concept in understanding SIPs aligns with contemporary research on the impact of childhood trauma and the use of substances as maladaptive coping mechanisms (88, 135, 136). This dissociative response to trauma and subsequent substance use suggests a multifactorial etiology involving neurobiological, psychological, and socio-environmental dimensions. The concept of mental automatism, introduced by Gaëtan Gatian de Clérambault, provides a framework for understanding the onset and progression of exogenous psychoses. Mental automatism encompasses involuntary phenomena in motor, sensory, and cognitive domains, which are perceived as foreign and intrusive by the individual. This phenomenon aligns with the early stages of psychotic states, where disruptions in ideation processes precede the development of delusions and hallucinations. Our study highlights the role of mental automatism in substance-induced psychoses, where the introduction of psychoactive substances leads to significant cognitive and perceptual disturbances. These automatic phenomena often manifest as eidetic experiences, such as thought echoes and intrusive thoughts, which are not initially tied to delusional content but progressively influence the individual’s mental state. De Clérambault’s differentiation between minor and major automatism is particularly relevant in clinical practice, as it allows for the early identification of subtle psychotic symptoms before they escalate into full-blown delusions. This distinction emphasizes the importance of recognizing and addressing these early signs to prevent the progression of psychosis in individuals using NPS.

Our analysis has also revealed another psychopathological element of considerable clinical importance, namely the concept of temporality. Phenomenological studies have highlighted that temporality is a fundamental aspect of the psychic life of patients, which can assume various connotations in severe psychopathological experiences. The subjective experience of time is profoundly altered in individuals experiencing SIPs, leading to a distorted perception of reality and a fragmented sense of self. This disruption is linked to the dissociative and automatic phenomena induced by psychoactive substances, which interfere with the continuity of identity and temporal awareness, for instance, the tendency to fix a person to a single temporal point immobilized within the infinite expanse of a moment. Our findings suggest that the altered temporality in exogenous psychoses contributes to the severity and persistence of psychotic symptoms. Understanding this aspect of the clinical-existential experience is crucial for developing targeted interventions that address the specific temporal disruptions experienced by these patients (127). In recent years, marked by the rapid spread of new substances of abuse, the clinical psychiatrist must not overlook the knowledge of the main clinical characteristics of exogenous psychoses compared to endogenous forms. It is essential to grasp the value of phenomenological psychopathology not only as a training itinerary for the psychiatrist but also as a foundational element of clinical psychiatric practice. Its primary declination is to instruct the psychiatrist in a specific mental attitude connected to the method that allows the acquisition of psychopathological knowledge. This approach can tend toward at least three directions:

-The search for higher-level psychopathological organizers: By broadening the horizon in which the reception of individual phenomena occurs, these organizers ultimately represent a donation of meaning to the phenomena themselves. The elements we analyzed (dissociation, automatism, and temporality), for example, are generators of psychopathological situations. This expanded understanding can provide a more comprehensive framework for interpreting and addressing these conditions.

-Theoretical foundations and mental attitude toward exogenous psychoses: the second direction of possible development of phenomenological psychopathology, which directly descends from the first, is to establish the theoretical premises and a mental attitude toward approaching the world of exogenous psychoses, which are often neglected or underestimated. Clinically, recognizing early dissociative and automatic phenomena in patients with substance use is vital for timely interventions aimed at preventing the progression to chronic psychotic states. Furthermore, understanding the unique temporal distortions in SIPs can guide the development of targeted therapeutic strategies that restore continuity in the patients’ lived experience of time.

-Intersection with biological research: particularly concerning the interface between phenomenological observations and neuroscientific findings. This integration is exemplified by contemporary neurobiological frameworks, notably the REBUS (RElaxed Beliefs Under pSychedelics) model proposed by Carhart-Harris and Friston (137). Their paradigm suggests that psychedelic substances induce temporary perturbations in hierarchical predictive processing networks, resulting in a systematic relaxation of high-level prior beliefs. This mechanism facilitates the emergence of novel states of consciousness, which, while potentially destabilizing, provide valuable insights into the architecture of conscious experience. The convergence of phenomenological and neurobiological approaches creates a fertile theoretical ground where subjective experiential data can be meaningfully correlated with underlying neural mechanisms. This methodological synthesis advances our understanding of psychiatric disorders by bridging the explanatory gap between first-person experience and third-person neurobiological observations, potentially illuminating the neural correlates of altered states of consciousness and psychopathological phenomena.

In conclusion, the integration of phenomenological psychopathology into clinical psychiatry serves as a comprehensive approach that enhances the psychiatrist’s ability to interpret and treat both exogenous and endogenous psychoses. By fostering a deeper understanding of the underlying psychopathological mechanisms and their neurobiological correlates, this approach promotes a more effective and holistic clinical practice.
